# Brainstem levels of transcription factor AP-2 in rat are changed after treatment with phenelzine, but not with citalopram

**DOI:** 10.1186/1471-2210-5-1

**Published:** 2005-01-21

**Authors:** Cecilia Berggard, Mattias Damberg, Lars Oreland

**Affiliations:** 1 Department of Neuroscience, Unit of Pharmacology, Uppsala University, PO Box 593 BMC, SE-751 24, Uppsala, Sweden

## Abstract

**Background:**

Before therapeutic effect is obtained after treatment with antidepressant drugs, like serotonin selective reuptake inhibitors (SSRIs), tricyclic antidepressants (TCAs) and monoamine oxidase inhibitors (MAO-Is) there is an initial lag-period of a few weeks. Neuronal adaptations on a molecular level are supposed to be involved in the initiation of the antidepressant effect. Transcription factor AP-2 is essential for neuronal development and many genes involved in the brainstem monoaminergic systems have binding sites for AP-2 in their regulatory regions. The genotype of the AP-2β isoform has been associated with e.g. anxiety-related personality traits and with platelet MAO activity. In addition, previous studies have shown that the levels of AP-2α and AP-2β in rat whole brain were decreased after 10 days of treatment with citalopram (SSRI) and imipramine (TCA), and were increased with phenelzine (MAO-I).

**Results:**

In the present study, we report that treatment with citalopram for 1, 7 or 21 days did not have effect on the AP-2 levels in rat brainstem. However, after treatment with phenelzine for 1, 7 or 21 days the levels of AP-2α and AP-2β had increased after 7 days, but had returned to control levels at day 21.

**Conclusion:**

The decrease in AP-2 levels in rat whole brain previously seen after treatment with citalopram does not seem to be localised to the brainstem, it may rather occur in the monoaminergic terminal projection areas. The present data suggest that the increase in AP-2 levels previously seen in rat whole brain after subchronic treatment with phenelzine is located in the brainstem. It cannot, however, be excluded that other brain regions are involved.

## Background

There is a large number of pharmacological strategies for treating serotonin-related disorders like depression and anxiety. Well-known antidepressant drug effects are blockade of the serotonin (5-HT) and/or norepinephrine (NE) reuptake pumps, direct effects on the 5-HT- and/or NE receptors and inhibition of the monoamine oxidase A (MAO-A) enzyme. Irrespective of which drug or combination of drugs that is used, there is a delay of a few weeks before any therapeutic effect is noticed. This initial lag-period usually is associated with several side-effects, many of which fade away with the appearance of the therapeutic effect. Considering the lag-period needed for the antidepressant effect to emerge, it has been suggested that the antidepressant mechanisms involve secondary molecular neuronal adaptations, rather than being a result of the primary actions of the drugs [1,2]. Some attempts to elucidate the molecular mechanisms involved in such lag-period have been reported, for example both SSRIs and MAO-Is cause desensitization of somatodendritic 5-HT_1A _autoreceptors [3,4]. Furthermore, SSRIs have also, during the initial lag period, been shown to cause desensitization of terminal 5-HT_1B/1D _autoreceptors [5,6]. Gaining more knowledge about molecular mechanisms involved in the initial lag-period may prove important for the discovery of new antidepressant drug targets and also for the knowledge of the mechanisms of action of antidepressants. Transcription factors, with their specific ability to regulate gene expression, have been suggested as prominent novel drug targets [7-11]. Transcription factor AP-2 is a critical regulatory factor for neuronal gene expression and neuronal development, e.g., in the brainstem [12-14]. Five different AP-2 genes have been identified, i.e., AP-2α, AP-2β, AP-2γ, AP-2δ and AP-2ε [15-19]. The isoforms are expressed from different genes and have a molecular weight of around 50 kD. The cis-acting DNA sequences 5'-(G/C)CCCA(G/C)(G/C)(G/C)-3' and the palindromic sequence 5'-GCCNNNGGC-3' are considered as consensus AP-2 binding sites for all AP-2 proteins [20]. Several genes encoding proteins involved in the brainstem monoaminergic systems have multiple AP-2 binding sites in their regulatory regions [20-26] indicating an involvement of AP-2 in the expression of these genes. We have recently reported positive correlations between brainstem AP-2α and AP-2β levels and monoaminergic activity in rat frontal cortex [27], indicating a regulatory function of AP-2α and AP-2β not only for neuronal development, but also for neuronal adaptive mechanisms in the adult brain. In two independent studies it was shown that the AP-2β genotype is associated with anxiety-related personality traits [28,29]. The AP-2β genotype has also been linked to binge-eating disorder [30] and to platelet MAO activity [31], which the latter is associated with personality traits. Furthermore, the AP-2β genotype has been associated to CSF-levels of homovanillic acid (HVA) in women [32].

In a previous study, we reported that the levels of AP-2α and AP-2β were decreased in rat whole brain after treatment for 10 days with citalopram, imipramine and lithium, respectively [33]. We have also reported that citalopram changes the levels of AP-2α and AP-2β in rat whole brain in a time-dependent manner, i.e., AP-2 levels were decreased after 7 days of treatment but returned to control levels after 21 days of treatment [34]. Furthermore, AP-2α and AP-2β levels were shown to be increased in rat whole brain after 10 days of treatment with phenelzine [35]. In the present study, we report that neither treatment with citalopram for 1, 7 nor 21 days affect the AP-2α and AP-2β levels in the rat brainstem. Treatment with phenelzine, however, increased the levels of both AP-2α and AP-2β in the rat brainstem after 7 days of treatment, but after 21 days of treatment the levels had returned to control levels.

## Results

The mean relative amounts of AP-2α and AP-2β protein ± standard deviation (SD) for each of the citalopram-, phenelzine- and saline treated animal groups are shown in tables 1 and 2, respectively. No significant differences in the amounts of AP-2α and AP-2β were found between any of the citalopram and saline treated groups. With regard to phenelzine treatment, there was a significant increase in AP-2α levels after 7 days of treatment (2.74 ± 0.57 vs 2.16 ± 0.14: mean relative amount of AP-2α ± SD, phenelzine vs saline, p = 0.037), which returned to control levels after 21 days. A similar result was obtained with regard to AP-2β levels with a significant increase only after 7 days of treatment (2.25 ± 0.25 vs 1.86 ± 0.22: mean relative amount of AP-2β ± SD, phenelzine vs saline, p = 0.017).

**Table 1:** Relative amount of AP-2α protein ± SD, for the different animal treatment groups.

	**day 1**	**day 7**	**day 21**
**saline**	2.29 ± 0.43	2.16 ± 0.14	2.45 ± 0.33
**phenelzine**	2.28 ± 0.61	2.74 ± 0.57 *	2.54 ± 0.43
**citalopram**	2.35 ± 0.31	2.33 ± 0.49	2.49 ± 0.47

Values are means ± SD, for each group of animals, n = 6. *p < 0.05 as compared to animals treated with saline for the same time-period.

**Table 2:** Relative amount of AP-2β protein ± SD, for the different animal treatment groups.

	**day 1**	**day 7**	**day 21**
**saline**	2.00 ± 0.44	1.86 ± 0.22	2.10 ± 0.14
**phenelzine**	2.14 ± 0.61	2.25 ± 0.25 *	2.00 ± 0.28
**citalopram**	2.16 ± 0.34	2.07 ± 0.47	2.05 ± 0.28

Values are means ± SD, for each group of animals, n = 6. *p < 0.05 as compared to animals treated with saline for the same time-period.

When comparing the untreated naive animals with the treated animal groups no differences with regard to the levels of AP-2α (untreated animals: 2.49 ± 0.71, mean relative amount AP-2α ± SD) or AP-2β (untreated animals: 2.04 ± 0.64, mean relative amount AP-2β ± SD) were found. Moreover, no differences in the levels of AP-2α and AP-2β were observed between the groups of animals treated with saline for the different time periods.

## Discussion

In two independent studies, we have shown that the levels of AP-2α and AP-2β in rat whole brain were decreased after subchronic (7 or 10 days) treatment with citalopram [33,34]. We have also shown that the AP-2α and AP-2β levels in rat whole brain were increased after treatment with phenelzine for 10 days [35]. For several reasons, we hypothesized that the brainstem should be of particular importance in this regard. Thus, many genes encoding proteins involved in the brainstem monoaminergic systems have binding sites for AP-2 in their regulatory regions. Furthermore, we have previously observed correlations between brainstem AP-2 levels and cortical monoamine activity [27]. The lag-period initially seen before the antidepressant therapeutic effect is obtained also made it interesting to study possible changes in AP-2 levels over time. In the present study, we found that treatment with citalopram for 1, 7 or 21 days did not have any effect on the brainstem levels of AP-2α and AP-2β. Treatment with phenelzine for 7 days, on the other hand, increased the brainstem levels of AP-2α and AP-2β, but after 21 days of treatment the levels had returned to control levels. The phenelzine data presented here, are in line with our previous study showing an increase in AP-2α and AP-2β levels in rat whole brain after 10 days of treatment with phenelzine [35].

The different responses on AP-2 of citalopram and phenelzine are likely to be explained by the different molecular mechanisms of the two drugs. Considerering the specific drug targets for citalopram and phenelzine, respectively, the target for citalopram, 5-HTT, is specific for membranes of the serotonergic system, while the target for phenelzine, MAO-A, is not located in the serononergic neurons [36]. It has been shown that some SSRIs, to some extent, are also able to inhibit MAO-activity [37]. However, all SSRIs, including citalopram, tested had a much higher selectivity for MAO-B than MAO-A and MAO-A, and not MAO-B, is the enzyme considered to be involved in the antidepressant effect [38]. Thus, a MAO-inhibiting effect of citalopram should not be a confounding factor with regard to interpretation of the present results. The fact that citalopram treatment did not affect AP-2 levels in rat brainstem indicates that the decrease in AP-2 levels previously seen in rat whole brain after citalopram treatment, takes place in some other brain region. It has been shown that postsynaptic 5-HT_1A _receptors in hippocampus get an enhanced response to 5-HT after treatment with TCA (that blocks both 5-HT and NE reuptake) for a time-period that corresponds to the time required for initiation of the antidepressant therapeutic effect [39]. An increased 5-HT responsiveness has also been demonstrated for other serotonin receptors than 5-HT_1A _and in other projection areas than hippocampus [40]. Thus, there are reasons to presume that the effect we have seen on AP-2 levels after subchronic citalopram treatment occurs in the 5-HT projection areas rather than in the 5-HT cell bodies in the brainstem. With regard to the increase in brainstem levels of AP-2 after 7 days of phenelzine treatment, it is in line with previous reports that MAO-Is partially enhance the 5-HT transmission by increasing the amount of 5-HT released per action potential [38], an effect which is likely to be regulated by presynaptic mechanisms located in the brainstem. The temporary changes in AP-2 levels after administration of antidepressants (present data and [34]), coinciding in time with the appearance of side-effects, makes it tempting to speculate that those two phenomena are somehow interrelated.

In a previous study, we reported higher levels of AP-2α and AP-2β in rat whole brain in untreated naive animals compared to animals treated with citalopram or saline for different time periods [34]. In the present study, however, we did not see any differences in the brainstem levels of AP-2α and AP-2β between naive untreated animals and treated animal groups. This indicates that the changes in AP-2α and AP-2β levels in rat whole brain previously seen in animals treated with citalopram or saline compared to naive untreated animals are located in some other AP-2 containing brain region than the brainstem.

As mentioned earlier, we have previously shown that the AP-2β genotype is associated with platelet MAO activity [31]. Thus, it is seems likely that AP-2 is involved in the regulation of the expression of the MAO enzyme. A possible explanation for the elevated AP-2α and AP-2β levels during subchronic phenelzine treatment could be that they are part of a feedback mechanism to counteract the reduction in MAO activity.

## Conclusions

Unraveling of the molecular mechanisms involved in the initial phase of antidepressant treatment is essential for the development of new efficient antidepressant drugs with less side-effects. We find transcription factors, such as AP-2, with ability to regulate expression of specific genes involved in the monoaminergic mechanisms, to be interesting candidates as novel antidepressant drug targets.

## Methods

### Animals and treatment paradigms

Adult male Sprague-Dawley rats (10 weeks of age, B&K Universal AB, Sollentuna, Sweden) were housed in groups of three and maintained on a 12 hour light /dark cycle with food and water freely available. Animals were administered phenelzine (n = 18, 10 mg/kg, Sigma, Sweden), or citalopram (n = 18, 10 mg/kg, Lundbeck AB, Helsingborg, Sweden) subcutaneously with daily injections. All drugs were dissolved in saline (NaCl, 9 mg/kg). Saline treated animals (n = 18) received saline injections in the same volume as that given the citalopram and phenelzine treated animals. Each group of animals was treated for 1, 7 or 21 days, respectively. All animals were sacrificed by CO_2 _inhalation 24 hours after their last injection. A group of untreated naive animals (n = 6) was sacrificed after 21 days. After sacrifice the brainstem was dissected and nuclear extracts were prepared for measurement of AP-2 levels by Enzyme-Linked Immunosorbent Assay(ELISA). This study was carried out with permission from the local animal ethics committee in Uppsala, Sweden.

### Extraction of nuclear extracts

Rat brainstem was homogenized in 3 ml buffer A (10 mM HEPES, 10 mM KCl, 0.1 mM EDTA, 0.1 mM EGTA, 1 mM DTT, 0.5 mM PMSF, pH 7.9). The homogenate was incubated on ice for 15 minutes. To this 125 μl Nonidet P40 was added, and the homogenate was centrifuged for 30 seconds at 14000 rpm in 4°C. The pellet was resuspended in 500 μl buffer C 20 mM HEPES, 0.4 M NaCl, 1 mM EDTA, 1 mM EGTA, 1 mM DTT, 1 mM PMSF, pH 7.9). Thereafter the tubes were put on a shaker for 15 minutes and centrifuged at 14000 rpm for 5 minutes (4°C). The supernatant i.e. the nuclear protein were aliquoted and stored at -80°C. The protein concentration for all nuclear extracts were determined by the method by Lowry at al. (1951) [41]. The concentration of nuclear extracts were ~8 μg/μl.

### ELISA measurements

96-well microtiter plates were coated (50 μl/ well) with nuclear extracts (10 μg/ml) diluted in 50 mM Carbonate-Bicarbonate buffer pH 9.0. The plates were covered with parafilm and incubated overnight at 4°C. Antigen solution was then removed and 200 μl blocking buffer (PBS, 1 % BSA) was added to each well and the plates were incubated for two hours in room temperature. Following this the blocking buffer was removed and the plates were washed with PBS. Primary antibody (50 μl, goat polyclonal AP-2α and AP-2β, 15 μg/ml respectively, SDS Biosciences, Falkenberg, Sweden) diluted in blocking buffer was then added and the plates incubated overnight at 4°C. After incubation the antibody was removed and the plates were washed three times with Wash buffer I (PBS, 0.05 % Tween-20). Secondary antibody (Donkey anti-goat IgG AP conjugated, SDS Biosciences, Falkenberg, Sweden) diluted 1:350 in blocking buffer, was then added (50 μl) to each well and the plates were incubated for two hours in room temperature. After removal of the secondary antibody the plates were washed three times with Wash buffer I, and once with Wash buffer II (10 mM diethanolamine, 0.5 mM MgCl_2_, pH 9.5). Thereafter, 50 μl substrate (Phosphatase substrate, 5 mg tablets, Sigma, Sweden, diluted in 5 ml Wash buffer II) was added to each well. The reaction continued for 30 minutes and was terminated by adding 50 μl of 0.1 M EDTA, pH 7.5. The plates were analysed in an ELISA reader (Molecular Devices, Thermo Max) at optical density (OD) 405/490. The OD of the AP-2 isoforms for each rat was correlated to a value in a standard curve, where known concentrations of antibody were plotted against optical density. The value form the standard curve was then divided with the concentration of total protein in the nuclear extracts. The quota was used as a value of the relative amount of AP-2α and AP-2β protein. Each rat were analysed twice for accuracy.

### Statistical analyses

The statistical comparisons between drug treated and saline treated animals for each time-point were analysed using unpaired t-test. When comparing the groups of untreated animals with all treatment groups we used analysis of variance (ANOVA) and Fisher's Protected Least Significant Difference (PLSD). To test if any of the groups of saline treated animals differed in the amounts of AP-2α and AP-2β protein ANOVA and PLSD test were used. All calculations were performed using Stat View 5.0 software (SAS Institute Inc., Cary, NC, USA). Results have been considered statistically significant when p < 0.05.

## Authors' contributions

CB planned the experiments, carried out the molecular and statistical analyses and drafted the manuscript, MD participated in planning the experiments and writing of the manuscript, LO conceived of the study, and participated in its design and coordination. All authors read and approved the final manuscript.
